# Insect Bio-inspired Neural Network Provides New Evidence on How Simple Feature Detectors Can Enable Complex Visual Generalization and Stimulus Location Invariance in the Miniature Brain of Honeybees

**DOI:** 10.1371/journal.pcbi.1005333

**Published:** 2017-02-03

**Authors:** Mark Roper, Chrisantha Fernando, Lars Chittka

**Affiliations:** 1 Biological and Experimental Psychology, School of Biological and Chemical Sciences, Queen Mary University of London, London, United Kingdom; 2 Google DeepMind, London, United Kingdom; 3 School of Electronic Engineering and Computer Science, Queen Mary University of London, London, United Kingdom; Universität Bielefeld, GERMANY

## Abstract

The ability to generalize over naturally occurring variation in cues indicating food or predation risk is highly useful for efficient decision-making in many animals. Honeybees have remarkable visual cognitive abilities, allowing them to classify visual patterns by common features despite having a relatively miniature brain. Here we ask the question whether generalization requires complex visual recognition or whether it can also be achieved with relatively simple neuronal mechanisms. We produced several simple models inspired by the known anatomical structures and neuronal responses within the bee brain and subsequently compared their ability to generalize achromatic patterns to the observed behavioural performance of honeybees on these cues. Neural networks with just eight large-field orientation-sensitive input neurons from the optic ganglia and a single layer of simple neuronal connectivity within the mushroom bodies (learning centres) show performances remarkably similar to a large proportion of the empirical results without requiring any form of learning, or fine-tuning of neuronal parameters to replicate these results. Indeed, a model simply combining sensory input from both eyes onto single mushroom body neurons returned correct discriminations even with partial occlusion of the patterns and an impressive invariance to the location of the test patterns on the eyes. This model also replicated surprising failures of bees to discriminate certain seemingly highly different patterns, providing novel and useful insights into the inner workings facilitating and limiting the utilisation of visual cues in honeybees. Our results reveal that reliable generalization of visual information can be achieved through simple neuronal circuitry that is biologically plausible and can easily be accommodated in a tiny insect brain.

## Author Summary

We present two very simple neural network models based directly on the neural circuitry of honeybees. These models, using just four large-field visual input neurons from each eye that sparsely connect to a single layer of interneurons within the bee brain learning centres, are able to discriminate complex achromatic patterns without the need for an internal image representation. One model combining the visual input from both eyes showed an impressive invariance to the location of the test patterns on the retina and even succeeded with the partial occlusion of these cues, which would obviously be advantageous for free-flying bees. We show that during generalization experiments, where the models have to distinguish between two novel stimuli, one more similar to a training set of patterns, that both simple models have performances very similar to the empirical honeybee results. Our models only failed to generalize to the correct test pattern when the distractor pattern contained only a few small differences; we discuss how the protocols employed during training enable honeybees to still distinguish these stimuli. This research provides new insights into the surprisingly limited neurobiological complexity that is required for specific cognitive abilities, and how these mechanisms may be employed within the tiny brain of the bee.

## Introduction

Honeybees (*Apis mellifera*) display an impressive visual behavioural repertoire as well as astounding learning capabilities. Foragers rely on visual and olfactory cues identifying rewarding flowers. Being able to recognise informative cues displayed by flowers can be assumed to facilitate fast and efficient decision-making. Indeed, honeybees can be trained to discriminate by an impressive range of visual cues; symmetry [1–3], arrangements of edges [4–6], size [7, 8], pattern disruption [9] and edge orientation [10–12]. These abilities are all the more impressive since trained bees are able to apply these same learnt cues to patterns which may have little or no resemblance to the original training patterns, so long as they fall into the same class of e.g. plane of symmetry, or edge orientation.

This rich visual behaviour despite a relatively tiny brain makes honeybees an ideal model species to explore how visual stimuli are processed and to determine if generalization requires a complex neuronal architecture. Using the published intracellular recordings of large-field optic ganglia neurons to achromatic stimuli [13, 14] and the known anatomical morphologies of mushroom body (learning centres) class II ‘clawed’ Kenyon cells [15] we designed two simple, but biologically inspired models. These models were not created, or indeed in any way ‘tweaked’ to replicate performance at any particular visual task. Instead they attempt to explore how well, or poorly, the known neuronal types within the bee brain could solve real behaviourally relevant problems and how much neuronal complexity would be required to do so. The initial models presented here were therefore kept very basic with limited neuronal pathways and very simple synaptic connections from the optic lobes to the mushroom bodies. In addition, to comprehend how these optic lobe neuron responses alone may explain the bees’ discrimination abilities and behavioural performance, we did not employ any form of learning in these models. Since two of the optic ganglia (medulla and lobula) of bees extend a variety of axonal fibres to both the ipsilateral and the contralateral mushroom bodies and, as opposed to axons from different regions of the optic lobes that are distinctly layered within the mushroom bodes, there is no apparent segregation of the visual inputs from the individual corresponding left and right eye regions [16, 17], we tested the discrimination and generalization performance difference between retaining independent inputs from each eye and combining the neuronal input from both eyes within our simulated mushroom body models. These models allowed us to simulate achromatic pattern experiments and compare the simulation performances of our two different bee-brain models—henceforth called ‘simulated bees’, to the performance of actual honeybees in these same specific experiments.

We drew on twenty-four experiments from three published honeybee behaviour papers [18–20] providing results on both the discrimination abilities of free flying bees perceiving complex bar and spiral patterns from a distance, and their generalization abilities while fixating, slow hovering scans 1–5cm in front of presented patterns.

The surprising ability of one of our extremely simple simulated bees to discriminate patterns correctly even with the partial occlusion of the test stimuli, its invariance to the location of the visual cues on the eyes, and generalization performances almost identical to real bees, provides new insights into the relationship between behaviour complexity and its neural circuitry underpinnings, significantly contributing to our understanding of the fundamental requirements needed for specific cognitive abilities.

## Results

To evaluate the performance of our models, we simulated the theoretical responses of mushroom body Kenyon cells [16, 21] to a range of achromatic patterns previously used in honeybee behavioural experiments [18–20]. These particular experiments were selected primarily because of the complexity of the patterns used, having variation in both the orientation and length of the edges within small regions of the patterns. In addition, the chosen experiments provided a broad range of behavioural results, including tasks bees found difficult or impossible to solve, and tasks with over 80% correct pattern selections.

Sensory input for our models was generated based on the known neuronal responses of lobula (3rd optic ganglion) large-field orientation-sensitive neurons discovered in insects [13, 14]. These intracellular tuning curve recordings allowed us to calculate the theoretical responses of eight lobula orientation-sensitive neurons (a type A and a type B neuron from the upper and lower region of each eye) for each of the required experiment patterns. The firing rate responses of these neurons were subsequently passed as inputs to the appropriate mushroom body models’ Kenyon cells.

Given the apparent non-retinotopic distribution of visual inputs from the corresponding left and right eye regions in the bee mushroom bodies [16], we created two models, which we call “DISTINCT” and “MERGED”, to explore the effect of segregating or merging synaptic connections from the lobula orientation-sensitive neurons originating from left and right eyes onto individual Kenyon cells within the mushroom bodies.

**Fig 1:**
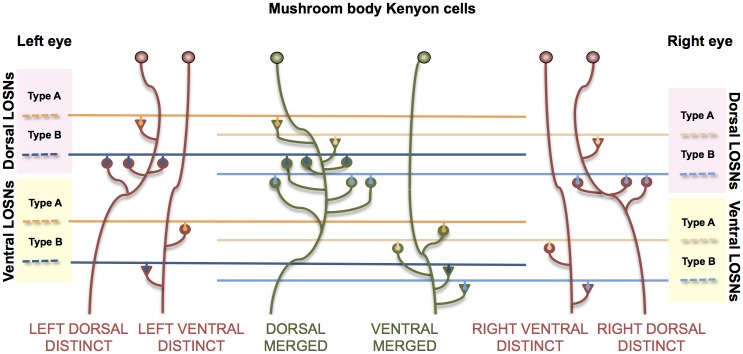
Schematic representation of DISTINCT and MERGED models. Representation of how the lobula orientation-sensitive neurons (LOSN) connect to each models’ Kenyon cells. The DISTINCT model’s Kenyon cells (red neurons) receive LOSN inputs from just **one** quadrant of the visual field, either the dorsal or ventral half of the left or right eye. In this example the dorsal Kenyon cells each have an inhibitory (triangle) LOSN type A synapse and three LOSN type B excitatory (circle) synapses (see Methods: Table 1 type 046). The dorsal DISTINCT Kenyon cells in this example each have one excitatory type A and one inhibitory type B synapse (see Methods: Table 1 type 001). The MERGED model Kenyon cells (green neurons) have the same configuration types as the respective dorsal and ventral DISTINCT neurons, but this model combines visual input originating from either the dorsal or ventral regions of **both** eyes; in the example the ventral MERGED neuron has one inhibitory connection from a type A LOSN and three excitatory LOSN type B synapses from the dorsal left eye and therefore must have the respective three excitatory type B and one inhibitory type A synapses from the ventral right eye.

The first of these models (DISTINCT) assumed that each Kenyon cell within the simulated bee’s brain would receive distinctly segregated lobula inputs originating from either the left or the right eye. This was further segregated into either the dorsal or ventral half of that visual field, as implicated in behavioural [12], neuroanatomical [16] and neurophysiological [17] studies. Fig 1 shows a schematic example of four of the DISTINCT model’s Kenyon cells (red neurons), these receive a variety of excitatory and inhibitory synaptic connections from type A and type B lobula orientation-sensitive neurons (see Methods), but each Kenyon cell has lobula neurons which all originate from either the dorsal or ventral region of just one eye.

The second model (MERGED) used the exact same lobula neuronal inputs but was designed such that its individual Kenyon cells would always receive input from both the left and right eyes. Again we assumed a distinct segregation of the dorsal and ventral halves of each eye’s visual field. To keep the model simple, and allow us to compare the respective models’ Kenyon cell responses, we established respective pairs of type A and type B orientation-sensitive neuronal inputs. This was done in such a way that if, for example, a Kenyon cell received an excitatory input from a type A originating from the ventral-left eye and an inhibitory type B synapses also from the ventral-left eye, it would have to have the respective excitatory type A synapses and an inhibitory type B synapse originating from the ventral-right eye lobula orientation-sensitive neurons (see Fig 1).

The above models allowed us to simulate the Kenyon cell responses to particular patterns. To assess the performance of these models solving discrimination or generalization problems, and to compare with the honeybee empirical results, we made the following assumption. The honeybee choice selection (which of two test patterns the bee would choose during an experimental evaluation trial) would be dependent on the similarity of that bee’s Kenyon cell responses to a learnt rewarding pattern presented during training trials, and the respective Kenyon cell responses to each of the subsequently presented test stimuli (similar to how olfactory learning in the mushroom bodies is thought to rely on the coincidence detection of Kenyon cell responses [22]). By this supposition, the honeybee should more often choose the test pattern that has the greater similarity of Kenyon cell responses to the rewarding training pattern, and furthermore the correct choice performance should depend on the relative difference of the two test patterns from this training pattern (see Methods).

All our simulations were therefore composed of three patterns; for each experiment’s simulation the rewarding pattern was the same as an original rewarding pattern presented to the honeybees during training. The correct test stimulus was the pattern that the real honeybees chose most often during the original behavioural evaluation trial and therefore should be “preferred” by our simulated bees also. The other test stimulus was therefore the incorrect pattern that the bees visited least often during their trials. The ratio of the differences in the Kenyon cell responses from the rewarding pattern to correct test pattern and rewarding pattern to incorrect test pattern produced an individual experimental simulation trial ‘Kenyon cell similarity ratio’. The average of these results over multiple simulation trials, per experiment, were used to produce each model’s simulated bee’s overall experimental performance (see Methods).

Thus, a simulated bee performance of approximately 50%, with individual simulation trial Kenyon cell similarity ratios of ≤0.5, meant that there was a similar or greater similarity from the rewarding pattern to the incorrect test pattern, rather than to the correct test pattern that the honeybees chose most often. This was therefore assumed to be our models’ equivalent of the bees’ inability to accurately discriminate or generalize to the test patterns. Similarly, an average Kenyon cell similarity ratio of ≥0.6, resulting in a simulated bee performance of ≥60% correct choices, indicated a greater Kenyon cell similarity between the presented rewarding stimulus and correct test patters than to the incorrect pattern, and therefore our simulated bees would be considered able to discriminate, or generalize to, the correct test pattern. These calculations allowed us to compare our DISTINCT and MERGED simulated bee performances to the respective honeybee correct choice percentages from the published literature [18–20].

### Experiment set 1: Discrimination

The ability to discriminate between visual patterns is essential for honeybees allowing them to identify familiar flowers and landmarks while navigating on foraging trips and locating the correct hive entrance upon their return. Nonetheless even for these types of precisely defined visual stimuli, some form of location invariance of a stimulus on the retinae would undoubtedly be required, as it is unlikely bees would perfectly align the stimulus against their eyes on every single flight in order to make a discrimination decision. Indeed it would be an undesirable necessity that they should have to do so.

**Fig 2:**
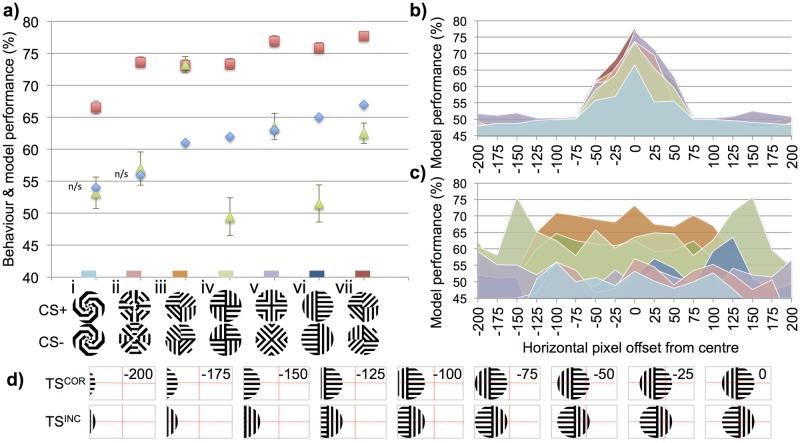
Model results for experiment set 1. Exemplary summary of honeybee behaviour and model performance for the discrimination tasks. In the behavioural experiments [18] different groups of honeybees were differentially trained on a particular pattern pair, one rewarding (CS+) and one unrewarding (CS-). **(a)** Blue diamonds: honeybee result, percentage of correct pattern selections after training. Red squares: performance accuracy of the DISTINCT simulated bee when test stimuli were presented in the centre of the field of view. Green triangles: performance accuracy of the MERGED simulated bee for the centralised stimuli. Error bars show standard deviation of the Kenyon cell similarity ratios (as a percentage, and centred on the simulated bee performance value; which was equivalent to average Kenyon cell similarity ratio over all simulation trials). Standard deviations were not available for the behaviour results. Small coloured rectangle on x-axis shows the corresponding experiment colour identifiers in (b, c). **(b, c)** Performance accuracy of the DISTINCT (b) and MERGED (c) simulated bees when comparing the rewarding patterns (CS+) with the corresponding correct (TS^COR^) and incorrect (TS^INC^) pattern pairs when these patterns were horizontally offset between 0 and ±200 pixels in 25 pixel increments (see d). Colour of region indicates the corresponding experiment in (a), performance at 0 horizontal pixel offset in (b), (c) is therefore also the same corresponding DISTINCT or MERGED result in (a) **(d)** Example of the correct and incorrect pattern images when horizontally offset by -200 pixels to 0 pixels, similar images were created for +25 pixels to +200 pixels. Experiment images were 300 x 150 pixels in size; patterns occupied a 150 x 150 pixel box cropped as necessary. Number in top right of each image indicates number of pixels it was offset by; these were not displayed in actual images. Red dotted lines show how pattern was subdivide into the dorsal left eye, dorsal right eye, ventral left eye and ventral right eye regions. Each region extended a lobula orientation-sensitive neuron of type A and a type B to the models’ mushroom bodies (see Fig 1). The DISTINCT simulated bee performs much better than the MERGED model’s simulated bee and empirical honeybee results when there is no offset in the patterns (a), but with only a small offset (±75 pixels) the DISTINCT simulated bee is unable to discriminate the patterns (b) whereas the simulated bee based on the MERGED model is able to discriminate most of the patterns over a large range of offsets (c).

To test our two models (DISTINCT, MERGED) for the effect of location of the stimuli within the visual field, we simulated the experiments of Zhang and Horridge [18] who explored the ability of freely flying honeybees to discriminate two large (24cm diameter) vertically displayed patterns composed of multiple oriented bars. For these experiments, a bee’s pattern choice was recorded when it approached within 27cm of either pattern (see [18] for apparatus description). Presuming that honeybees would learn the correct pattern features when feeding at, or being close to, the centre of a rewarding pattern, we first calculated our Kenyon cell responses to these same rewarding patterns. We next determined each of our simulated bees’ performance accuracies when any of the two given test stimuli patterns (correct pattern was identical to the rewarding pattern, the incorrect pattern was a rotated or mirrored version of this rewarding pattern) were offset horizontally between -200 pixels and +200 pixels in 25 pixel increments. A zero pixel offset would align the pattern perfectly in the centre of the field of view with half the pattern visible in each eye. Whereas a ±75pixel horizontal offset would remove the whole pattern from one eye’s visual field, and at ±200 pixels leave only a small portion of the pattern visible in just one eye (Fig 2).

With zero offsets of the correct and incorrect test patterns, we found that the DISTINCT simulated bee was able to discriminate all of the presented pattern pairs. Indeed, despite its simplicity, the model design allowed it to outperformed real honeybees whose best result was 67% compared to DISTINCT simulated bee’s 78% accuracy for the same pattern pair (Fig 2). This model bee also discriminated the two pattern pairs that real honeybees failed to discriminate (spiral patterns—bee: 53.7% *p*>0.7 *n* = 54 [18]–DISTINCT: 67%, octagonal patterns—bee: 56.4% *p*>0.2 *n* = 140 [18]–DISTINCT: 74%, see Fig 2).

The MERGED simulated bee results were far lower than the DISTINCT model’s discrimination accuracies but compared better to that of the experimental results. As with real honeybees’ behaviour, the MERGED simulated bee did not reliably discriminate the spiral and octagonal pattern, achieving simulation results of just 53% (bee: 54%) and 57% (bee: 56%) respectively. Out of the seven tested pattern pairs the only notable difference from the behavioural results was the MERGED bee’s inability to discriminate the two left / right reversed pattern pairs yielding only 49% and 52% respectively (Fig 2). Here honeybees achieved 62% and 65% in the behavioural experiments.

Clearly the simpler model (DISTINCT) returned more accurate discrimination results and outperformed both the more derived model (MERGED) and the honeybees. Our results raise the interesting question why the honeybees performed so poorly on some of the patterns, when a very simple model (DISTINCT) was easily able to discriminate the patterns while using just eight large-field orientation-sensitive neuronal inputs.

However, progressively offsetting the test patterns from the centre of the field of view revealed the lack of robustness of the DISTINCT model to cue variation. Here the simulation performances dropped much faster than that of the simulated bee using the MERGED model. In fact with as little as ±75 pixel offset (where the whole pattern was still visible) the performance of the DISTINCT simulated bee fell below 52% for all pattern pairs (Fig 2).

With the MERGED model, all discriminable patterns (>64% accuracy at 0 pixel offset) (still achieved accuracies above 60% when the patterns were offset by ±75 pixel. Even when these patterns were offset by as much as ±125 pixels rendering almost half of the patterns invisible the model’s lowest simulated performance for these experiments was 57%—i.e. markedly more than for the DISTINCT model. Beyond this offset distance, only the one pattern pair (crosses, see Fig 2) was effectively discriminated, at a level of ≥59% accuracy during simulations even when only small portions of the patterns were still visible.

Our results show that by simply combining inputs from both the left and right eyes onto mushroom body Kenyon cells, discrimination abilities are effectively freed of requiring perfect cue alignment on the retinae. Although this reduces the maximal discrimination accuracy, it allows for a much more robust and versatile employment of this cognitive tool in most realistic free flight navigation and resource locating scenarios.

### Experiment set 2: Generalization

Experienced honeybee foragers may identify rewarding flowers based on those features that most reliably predict reward amongst the available flower species. Honeybees able to generalize to this limited feature set would reduce the need to learn all the exact features (or indeed photographic templates) of each individual flower type visited and subsequently having to best-match these numerous complex templates when foraging on novel or less frequented floral resources [10, 20, 23].

**Fig 3:**
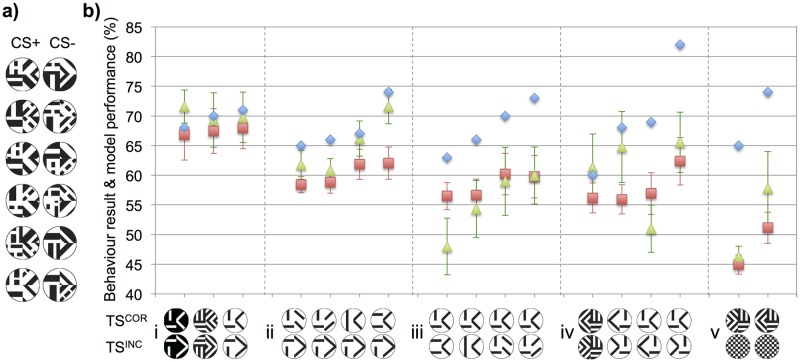
Model results for experiment set 2. Summary of honeybee behaviour and model performance for the generalization tasks. **(a)** The two sets of quadrant patterns (each set having similarly orientated bars in each quadrant of the pattern) that were used during the behavioural experiments [19, 20]). Honeybees were trained on random pairs of a rewarding pattern (CS+) and unrewarding pattern (CS-) selected from the two pattern sets, different groups of bees were tested on the reversal such that the CS- pattern would become the CS+ and vice-versa. **(b)** Blue diamonds: honeybee result, percentage of correct choice selections when tested with novel patterns of varying degrees of difference from the training patterns (here the correct pattern (TS^COR^) is the pattern the bees visited most often). Red squares: DISTINCT simulated bee performance when comparing each of the six rewarding patterns in a pattern set (a) against a novel test pattern pair (TS^COR^ and TS^INC^). Green triangles: MERGE simulated bee results for the rewarding pattern sets compared against each test pattern pair. Error bars show standard deviation of the Kenyon cell similarity ratios (as a percentage, and centred on the simulated bee performance value; which was equivalent to average Kenyon cell similarity ratio over the all simulation trials). Standard deviations were not available for the behaviour results. For simple generalisations (i) where the novel correct patterns had the similarly oriented bars to the rewarding pattern set and incorrect test pattern was similar to the unrewarding training patterns the DISTINCT and MERGED simulated bee performances were almost identical to those of the real honeybee results. For the harder generalisations; (ii) correct test pattern had one quadrant incorrect—the incorrect test pattern had all quadrants incorrect, (iii) correct pattern had all quadrants correct—incorrect pattern had three quadrants correct, (iv) mirror images and left-right reversals of the rewarding pattern layout, the simulated bee based on the DISTINCT model correctly generalised all pattern pairs but performed substantially worse than the real bees. The MERGED simulated bee failed most experiments in (iii) but did typically perform better than the DISTINCT bee in (ii) & (iv). Both simulated bees failed to generalise correctly if the correct pattern was a chequerboard, whereas real honeybees typically rejected this novel stimulus.

To explore these generalization abilities, Stach et al. [19, 20] trained honeybees on two sets of six patterns where within each set there were similarly orientated bars in each quadrant of the patterns (Fig 3). They then tested the bees’ ability to generalize from these training patterns to novel variations of the patterns. Unlike the previous experiments, these bees were able to fixate a small distance from the pattern before their final choice selection was recorded when they actually touched either of the two test patterns. For our simulations we therefore presented all the patterns in the centre of the field of view with zero horizontal, or vertical, offset applied, assuming this would be where a honeybee would make its final decision.

Fig 3 shows the experiments we simulated and the corresponding honeybee experimental results [19, 20]. The overall average difference from the simulation performance of all 17 generalization experiments to the corresponding empirical results for the DISTINCT model’s simulated bee was -9.83% and just -7.77% for the MERGED model’s simulated bee. However, as a direct correlation comparison of the model performances and behavioural results is not appropriate (see Methods), we followed the approach of the original studies [19, 20] and compared the model results against the experimental performances within smaller batches of similar generalization type tasks.

Our first batch of experiments, using patterns from Stach et al. 2004 [19], tested simple generalization from the training sets of six patterns to three novel pattern pairs. The experimentally preferred test stimulus patterns had bars orientated in the same direction as the corresponding quadrants of the rewarding training patterns, versus the incorrect distractor patterns with a similar visual style to the matching correct test pattern but with bars orientated in different directions to those of the rewarding pattern in each quadrant. We found that simulations of both the DISTINCT and MERGED models produced simulated bee results almost identical to the honeybee behavioural results (Fig 3). Both the percentage of honeybee correct choice selections for correct test patterns and our simulated bees’ performances were all between 67% and 72%.

Our second batch of experiments again followed the study of Stach et al. [19], here the correct patterns had three quadrants with correctly orientated bars and the final quadrant did not, the incorrect test patterns had incorrectly oriented bars in all four quadrants. The DISTINCT model achieved ≥58% throughout but performed typically 5–10% below the honeybees (Fig 3). The simulated bee based on the MERGED model outperformed the simulated bee of the DISTINCT model on all test pattern pairs with simulation performances ranging from 61–72%, once again extremely similar to that of the honeybee behavioural result range of 65–74%.

In our third batch of experiments utilizing the same Stach et al. dataset [19], the correct and incorrect test stimuli were very similar, the correct patterns having correctly oriented bars in all four quadrants and the incorrect patterns had just one quadrant with incorrectly oriented bars. Simulations of the MERGED model failed to allow its bee to generalize to the correct pattern in three out of four experiments, with individual simulation trials failing to achieve a Kenyon cell similarity ratio of more than 0.5 (Fig 3). The DISTINCT simulated bee managed to correctly generalize all of these patterns but with low accuracy of just 56% to 60%; the corresponding honeybee results ranging from accuracies of 63% to 73%.

Our fourth experiment set was compiled by taking test pattern pairs from the earlier work of Stach and Giurfa [20]. In this study, honeybees were presented with different combinations of either the original rewarding training pattern configuration, or the mirror image, or the left / right reversal of this layout. The DISTINCT model’s simulated bee was once again able to generalize correctly to all the experimental patterns (Fig 3). Although performing less well than real honeybees, the model showed similar lower generalization performances on the mirror image versus left-right patterns (56%) compared to that of the original rewarding pattern versus the mirror image patterns (62%). The MERGED simulated bee typically achieved higher accuracies that were more similar to the honeybee results than that of the DISTINCT model’s bee, correct generalization performances ranged from +1% to -12% different to the empirical result. Of note, the bees achieved a surprising 82% correct choice accuracy on one of these test pattern pairs almost 10% higher than any other task, our models had high results on this experiment (DISTINCT: 62%, MERGED: 66%) but we did not see these particular simulations outperform all others. Only two of the eight test pattern pairs (correct stimuli: original configuration, incorrect stimuli: left / right reversal) failed to generalize correctly with a performance of just 51% (individual simulation trial Kenyon cell similarity ratios ranging from 0.39 to 0.62 dependent on the particular pattern triplets presented) compared to the honeybee correct choice selection of 69%. During simulations both the DISTINCT and MERGED simulated bees showed a preference for the left / right reversal configuration compared to the mirror image pattern, they also preferred the correct configuration to the mirror image layouts, as did real honeybees (Fig 3).

The last of our experiment sets, again used patterns from Stach and Giurfa (2001) [20]. Fig 3 shows that both types of simulated bees were unable to generalize when presented with a chequerboard distractor pattern, with individual trial Kenyon cell similarity ratios as low as 0.4 (i.e. ‘preferring’ the incorrect pattern). Conversely, honeybees always preferred left / right or mirror image versions of the rewarding pattern configuration to that of the chequerboard option with behavioural results of 65% and 74% respectively.

Despite our models’ extreme simplicity, they largely predicted the honeybees’ generalization performances accurately for a majority of the tested pattern pairs. Our simulated bees did fail to generalize when the two test patterns were very similar (Fig 3). However, whereas honeybees were trained on both rewarding and unrewarding training patterns, our simulated bees only perceived the rewarding stimuli. This may account for some of the honeybees’ additional correct choice performance (see Discussion). Nonetheless, these results indicate that seemingly ‘complex’ tasks do not require advanced cognition. Instead, our DISTINCT and MERGED models provide evidence that visual pattern recognition and classification may in fact be the emergent properties of connecting just a small number of large-field visual inputs.

## Discussion

Apparently sophisticated cognitive abilities are often seen as a result of an equally complex neuronal architecture. However, here, this view is fundamentally challenged. Despite honeybees having a tiny brain consisting of less than one million neurons (as compared to eighty-six billion neurons in the human brain [24]), they still display an impressive range of cognitive abilities from learning to recognise pictures of human faces [25–27] to simple counting [28].

Using a modelling approach, we investigated how bees' ability to discriminate and generalize could be explained by simple neural networks. We have shown that for achromatic bar patterns, regularly used in honeybee behavioural experiments, bees may actually require very little sophistication in neuronal circuitry. The honeybee lobula orientation-sensitive neuron responses are thought [13, 29] to be the result of the summation of smaller receptive field orientation-sensitive neurons in the bee lamina or medulla (1^st^, 2^nd^ optic ganglia), similar to those found in other insect medullas [13, 14, 30–33]. This collation of smaller subunits allows the lobula orientation-sensitive neurons to encode a simplified summary of the oriented edges across the whole width of the bee eye. Although this means a bee cannot extract the exact retinotopic location or indeed orientation of individual edges through these neurons, our results show that, surprisingly, just eight of these large-field lobula neurons would be sufficient for the discrimination and generalization of the described patterns.

Our models also demonstrate, despite their simplicity, that just a single layer of simple connections from the lobula orientation-sensitive neurons to the mushroom body Kenyon cells would suffice to reproduce the empirical generalization results between a given rewarding pattern and the two test patterns. In fact our models may have had a more difficult challenge than that of real bees. During training the honeybees were exposed to both the rewarding patterns with a sugar water reward but also an unrewarding (water) or even aversive solution (quinine) on the training distractor patterns, this differential training would allow the bees to learn both those features consistent with reward but also those pattern features that were to be avoided. There is empirical evidence to show that choice accuracy as well as the pattern features learnt by bees are affected by the training regime (e.g. absolute conditioning (no distractor pattern) vs. differential conditioning [34, 35], and the penalty associated with a distractor [36–38]. Since it remains unclear how these different factors affect learning on the neuronal level, the theoretical models described here used very simple mathematics to calculate the similarity of the Kenyon cells responses to different stimuli, and from this produce theoretical simulated bee performances. Although this is very different to how learning would take place within the honeybee mushroom bodies, it did allow us to investigate how the lobula orientation-sensitive neuron responses alone may affect the honeybees’ performance during different discrimination and generalization experiments. In addition, it allowed us to study how different connections of the lobula neurons and Kenyon cells may also affect performance. Given our models employed no form of learning, it is all the more impressive that our simplified and experimentally disadvantaged simulated brains were able to generate largely similar results to actual bees.

The Kenyon cell outputs of our models were achieved solely by the summation of either excitatory or inhibitory connections from the lobula orientation-sensitive neurons (with predefined configurations, and fixed synaptic weights of either +1 or -1 respectively). These simulated Kenyon cell outputs allowed our simulated bees to discriminate and generalize the tested patterns with approximately 50% activation of their Kenyon cell populations (due to the reciprocal lobula orientation-sensitive neurons to Kenyon cell connection types, see Methods); we assumed, for comparison with our simple models, that some form of synaptic plasticity from the Kenyon cells to the mushroom body extrinsic neurons would allow the bees to associate the appropriate 50% active Kenyon cells to the rewarding training pattern, and from these adjusted synaptic weights make the behavioural decisions. However, neuronal recordings of the mushroom body lip, which receives olfactory input, shows just ~5% activation of the Kenyon cells mediated by a feedback inhibitory network in the mushroom body calyces [39]. It may be that when honeybees visit a correct pattern they can increase the firing rate or reduce the response latency of the Kenyon cells that fire, but potentially more importantly, may quiescent those Kenyon cells that incorrectly fired for the unrewarding, or punished, training pattern (during differential training). In this case the 5% of the Kenyon cells that are active (assuming the same value as for olfactory stimuli) would potentially be optimal to associate the rewarding stimulus with sucrose reward. Additional research is required to see if this greater specificity would actually account for some of the honeybees’ higher performance over that of our current models. It should be noted that ~50% of the olfactory projection neurons to the mushroom bodies are highly active when a particular odour is presented [40] providing a population coding response to a given odour, this differs considerably to that of the optic lobe neurons that typically have more specific firing rate tuning curve responses to particular stimuli. Due to issues with harnessing bees during visual learning tasks we currently lack the ability to record Kenyon cell responses for anything but the simplest visual stimuli (e.g. whole eye exposure to a single colour [41]). Unfortunately this means we do not yet have empirical evidence for the Kenyon cell activation level for visual stimuli. New research using walking bees in virtual reality rigs [42] may allow these activation levels, and Kenyon cell response changes, to be recorded during visual learning paradigms. These findings will undoubtedly provide vital information for the next generation of theoretical models, which could be used to understand the trial-by-trial learning process of bees.

Despite the limitations mentioned above, our simulated bees still performed almost identically to the real bees when making simple generalizations and only dropped in performance when either the test patterns began to differ from the oriented edges presented in the rewarding patterns or the correct and incorrect test patterns became very similar (Fig 3). Here the difference in the honeybees’ exposure to the unrewarding as well as rewarding stimuli during training almost certainly contributed to the typical 5–10% performance advantage compared to our simulated bees, which only used the rewarding stimuli. Again, future behavioural and electrophysiological research may reveal how training paradigms affect the learning on the neuronal level, which would allow corresponding adjustments to the new theoretical models.

During the offset pattern discrimination simulations (Fig 2) we found that simply combining the neuronal firing rates of lobula orientation-sensitive neurons from each eye onto individual Kenyon cells would allow for pattern discrimination with an impressive location invariance of the perceived stimuli. By merging information from both eyes, a very coarse representation of the whole 270° bee eye horizontal field of view can be produced. Surprisingly, this non-retinotopic representation appears sufficient to discriminate quite complex visual patterns, removing the need for the bees to have to store an eidetic or ‘photographic’ view of the pattern. As a pattern is offset from the centre of the field of view, such that it is visible in one eye more than the other (Fig 2), then the firing rates of all eight neurons (a type A and a type B lobula orientation-sensitive neuron in each of the four visual field regions—dorsal and ventral half of each eye) will adjust according to the oriented edges each region now perceives. With the DISTINCT model, as the pattern is offset the changes in the total synaptic input per Kenyon cell (compared to the zero offset pattern) are quite pronounced—as these are directly influenced by the amount the lobula neurons response change due to the addition, or removal, of oriented edges in each separate region. Therefore, the DISTINCT model’s discrimination ability is impaired the further a pattern is offset. In contrast, with the MERGED model, although the lobula-orientation sensitive neurons’ firing rates are the same as the DISTINCT model, by combining the lobula neuron responses from the left and right eyes even as the pattern is offset the total summated Kenyon cell values remain similar to the summated values with no offset (at least until the patterns begin to leave the field of view of both eyes). For example in the second generalization test the correct test stimuli had the orientation of bars in one quadrant of original rewarding pattern rotated through 90° (Fig 3); here the DISTINCT model had a whole quadrant producing incorrect Kenyon cell responses, whereas in the MERGED model only a proportion of the whole dorsal or ventral field of view is altered and thus a smaller number of Kenyon cells ‘misfire’.

**Fig 4:**
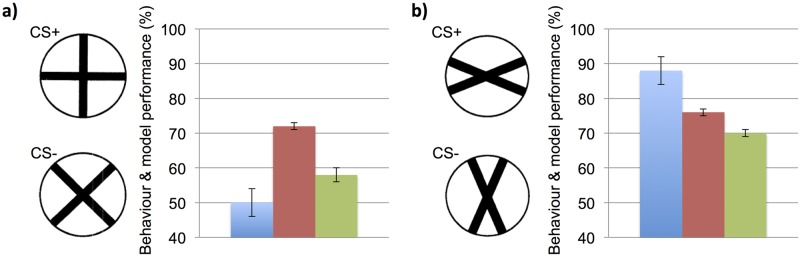
Model results for cross pattern experiments. Summary of honeybee behaviour and simulated bees’ performance for the discrimination of simple cross patterns. In the behavioural experiments [11] different groups of honeybees were differentially trained on a particular cross pattern pair, one rewarding (CS+) and one unrewarding (CS-). Blue: honeybee result, percentage of correct CS+ pattern selections after training. Red: performance accuracy of the DISTINCT simulated bee. Green: performance accuracy of the MERGED simulated bee. Behaviour Error bars for honeybee shows standard deviation. Error bars for models shows standard deviation of the Kenyon cell similarity ratios (as a percentage, and centred on the simulated bee performance value; which was equivalent to average Kenyon cell similarity ratio over the all simulation trials). **(a)** Discrimination of 90° cross and 45° rotation of this pattern. The DISTINCT simulated bee easily discriminates the patterns but honeybees cannot, the simulated bee based on the MERGED model achieved below 60% accuracy. **(b)** Discrimination of a 22.5° cross pattern and the same pattern rotated through 90°, both of the simulated bees and real honeybees can discriminate these cross patterns.

However, despite the typically good discrimination results over large offsets and the ability to discriminate when patterns are only partially visible, our results show that this mechanism may well come at the expense of discriminating certain types of stimuli. Complex spiral and octagonal patterns (Fig 2) were not reliably discriminated by our simulated bee based on the MERGED model or by real honeybees [18]. Surprisingly, honeybees have been shown unable to discriminate a very simple pair of 90° cross patterns (incorrect pattern rotated through 45°) [11] (Fig 4), despite their apparent differences to a human observer. Simulations of these experiments once again showed the MERGED model’s simulated bee’s closer similarity to the honeybee behavioural results, with a sub 60% discrimination performance on these simple cross patterns, whereas the DISTINCT simulated bee achieved over 70% accuracy. Interestingly both of the simulated bees, and honeybees, were able to discriminate a pair of 22.5° rotated cross patterns easily (incorrect pattern rotated through 90°) (Fig 4). It may well be that in allowing the neuronal architecture of the honeybee brain to overcome location variance for common stimuli, it has compromised its ability to discriminate specific, arguably less important cue combinations.

In a few specific instances our MERGED simulated bee failed to discriminate the tested pattern pairs, in contrast to the empirical results. The model’s inability to allow its simulated bee to discriminate the left / right reversal patterns in experiment four and six of the discrimination experiments (Fig 2) and experiment four of the generalization experiments (Fig 3) was no surprise as both the correct and incorrect test patterns presented the exact same orientations only in the reverse eyes, and hence produced the same summated input to the Kenyon cells, whereas the inability to discriminate the incorrect checkerboard pattern from the correct test patterns (Fig 3) may be down to the lack of a predominant orientation in this stimulus causing lobula orientation-sensitive neuron outputs which were equally dissimilar from the rewarding patterns as the correct test patterns confusing the system. It is most likely that in these experiments and while observing other similar stimuli the honeybees use other visual features (optic flow, symmetry, etc.) to which our very simple models did not have access. In addition, the poor concordance of the MERGED model simulated bee results and the honeybees in the generalization experiments may also result from the experimental paradigm that allowed the bees to fixate on the pattern at close range and make their final decision from a fixed perspective. This would, for these experiments, be very similar to the better-performing DISTINCT model’s simulated bee with zero stimuli offsets.

It is conceivable that honeybees have a combination of both DISTINCT and MERGED type lobula orientation-sensitive neuron to Kenyon cell configurations within their mushroom bodies. In this neuronally still simple scenario, attention-like processes could “selectively learn” the Kenyon cell responses that are good indicators of reward in a given experimental scenario. This might therefore account for some of the honeybees’ higher performance compared to that of our simulated bees based solely on the MERGED or DISTINCT models. Future work will investigate if there is an optimal distribution of distinct and merged lobula orientation-sensitive neuron connections to the Kenyon cells, or if synaptic plasticity is able to adjust the proportion of each connection type for a particular task. This modelling of bee visual processing and synaptic tuning may then be able to provide additional insights for machine vision applications where very lightweight computational solutions are required for object or landmark recognition, such as next generation self-drive vehicles and autonomous flight systems.

Our research shows that very simple neuronal connections, which would be easily accommodated within the miniature brain of a bee, are able to facilitate seemingly complex visual cognitive tasks. In addition the merging of visual information from both eyes, as seen in the mushroom bodies of bees [16], appears to be a very effective solution to partial occlusion and retinal location invariant pattern discrimination.

## Methods

### Calculating lobula neuronal responses

**Fig 5:**
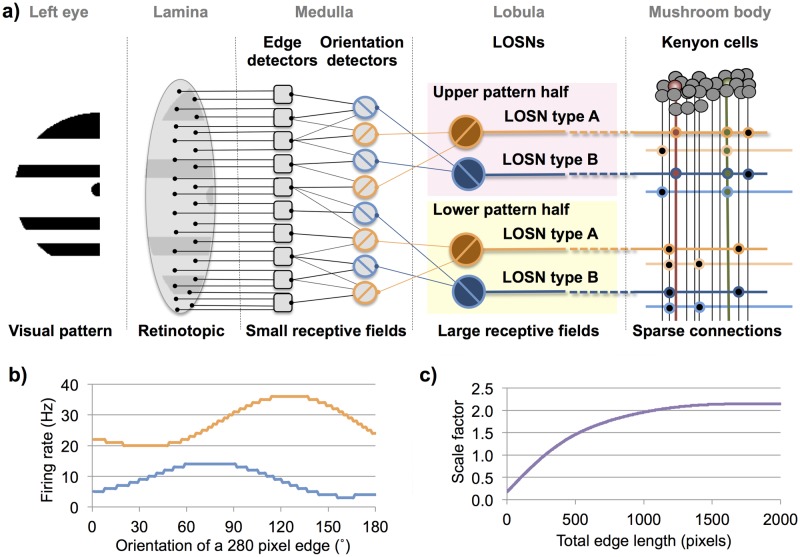
Schematic representation of the models. The pattern processing stages for the type A and type B lobula large-field orientation-sensitive neurons (LOSN) and their connectivity to the mushroom body Kenyon cells. **(a)** Each simulated eye perceives one half of the test image (left eye shown). Lamina: converts a given pattern image into a binary (black/white) retinotopic representation. Medulla: extracts edges resolvable by honeybees and determines the length of all orientations (0°-180°) within the upper and lower image halves. Lobula: within the upper and lower image regions, the LOSN firing rates for the type A and type B neurons are calculated (see Fig 6). The same process is repeated for the right eye producing in total eight LOSN responses. These are then passed to the appropriate 10,320 (DISTINCT model) or 5,160 (MERGED model) mushroom body Kenyon cells. **(b)** Firing rate responses of our theoretical LOSNs (type A: orange, type B: blue) to a 280 pixel edge at all orientations between 0°–180°; tuning curves adapted from honeybee electrophysiological recordings [13]. **(c)** Scale factor applied to the LOSN firing rates dependent on the total edge pixel length in each pattern quadrant, nonlinear scaling factor derived from dragonfly neuronal responses to oriented bars with differing bar lengths [14].

The simulated lobula (3^rd^ optic ganglion) large-field orientation-sensitive neurons used in our models were derived from the Yang & Maddess (1997) study on the honeybee (*Apis mellifera*) [13]. In these experiments, electrophysiological recordings where made from the lobula of tethered bees placed in front of CRT computer monitors; stimuli of oriented bars moving across one eye were presented at 30° angle intervals, in both the frontal and lateral eye regions. These neurons responded to the oriented bars moving anywhere across the whole width of the eye, but were maximally sensitive to orientations of 115° (type A) and 250° (type B) with angular half-widths of about 90°. We produced best-fit curves to both the reported type A and type B lobula orientation-sensitive neuron responses so that we could provide a theoretical neuronal response to a fixed 280-pixel edge at any orientation (Fig 5).

Bees presented with two identically oriented bars simultaneously in both the frontal and lateral regions of the eye generated lobula orientation-sensitive neuron responses that were higher than for a single bar in either eye region but less than the summated responses [13]. A similar nonlinear response was seen in dragonflies (*Hemicordulia tau*) [14] where the response to an oriented moving bar would increase with the length of the presented bar. Assuming that these honeybee lobula neuronal responses are due to a nonlinear summation of smaller orientation detectors in the lower lobula or medulla, we used this more detailed response curve recorded in the dragonfly to generate a best-fit scale factor curve for when the length of a presented edge increases (Fig 5). This allowed us to scale the lobula orientation-sensitive neuron responses for any oriented edge based on its length compared to the fixed length used for our LOSN tuning curves.

**Fig 6:**
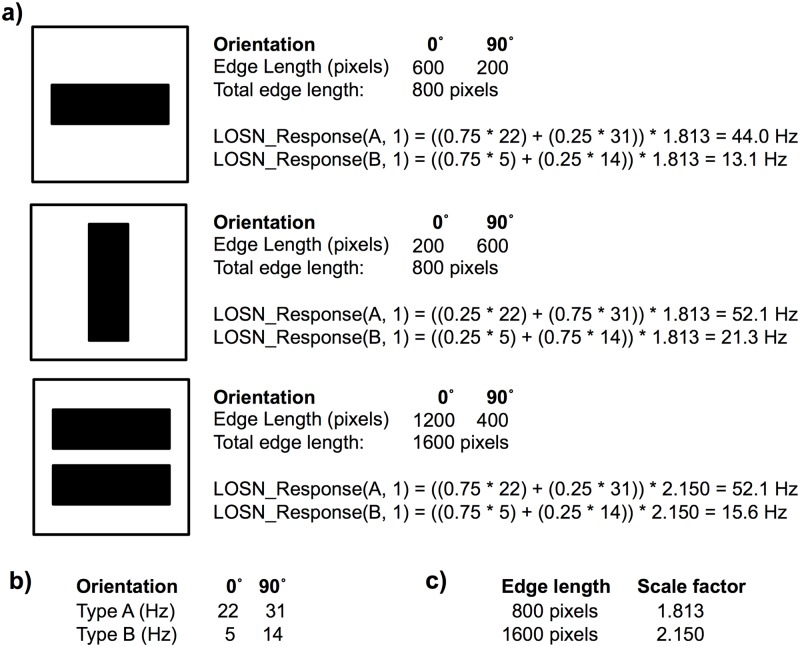
Worked example of LOSN calculations. Simplified example of the lobula orientation-sensitive neuron (LOSN) type A and type B firing rate response calculations. **(a)** Here we calculate values for just the left dorsal eye (quadrant 1) with only horizontal (0°) and vertical (90°) edges presented. In the single horizontal bar example (top) 75% of the overall edge length is at a 0° orientation (600 pixels out of total edge length of 800 pixels) and 25% of the edges at 90° orientations, thus the LOSN responses are influenced more by the response curve values at 0° than 90°. Conversely, the vertical bar is influenced more by the response curve values at 90°, resulting in overall higher LOSN firing rates. The two horizontal bars example (bottom) has the same proportion of orientations as the single horizontal bar (top). Although the total edge length is doubled, the LOSN firing rates are not twice as high; instead they are scaled using the non-linear scaling factor derived from dragonflies (see Fig 5 and Eq 1). Note that the LOSN type A firing rate is the same for a single vertical bar as it is for two horizontal bars (52 Hz). **(b)** LOSN type A and type B response curve values for 0° and 90° (see Fig 5). **(c)** LOSN scale factors for 800 and 1600 pixel edges (See Fig 5).

To account for multiple edges at different orientations in any one image, we again presume that the overall lobula orientation-sensitive neuron response is composed from smaller subunits in the medulla or early lobula and will vary with both the total length and abundance of all oriented edges within the receptive field that that neuron receives information from. We thus calculated the overall type A and type B responses for any given pattern using the edge length histogram datasets for all four quadrants of that pattern (see below). For each quadrant and each lobula orientation-sensitive neuron type, we summated the proportion (orientation edge length / total edge length) of each edge orientation (0°-180°) and multiplied it by the neural response for that orientation on our standard 280 pixel edge curve (Fig 5). This total value was then corrected by the scaling factor derived from the total edge length within that quadrant (Fig 5). This produced a type A and type B response (Eq 1) for each quadrant of the visual field (see Fig 6) and therefore eight lobula orientation-sensitive neuron responses in total for a given pattern. These image specific responses were saved with the pattern’s unique identification number (UID) and subsequently used as the sensory inputs to the Kenyon cells of our mushroom body models.

$$LOSN\_Response\left(x,q\right)=\left(\overset{180}{\underset{i=1}{\sum }}\left(\frac{H\left(q,i\right)}{\sum H\left(q\right)}*C\left(x,i\right)\right)\right)*S\left(\sum H\left(q\right)\right)$$ (1)

Where LOSN: lobula orientation-sensitive neuron; x: LOSN type A or type B; q: visual field quadrant 1:4; H: matrix of edge lengths for each orientation (1° increments) in each quadrant; C: response of LOSN type x to a 280 pixel edge at orientation; S: scale factor for given total edge length (Fig 5).

### Calculating mushroom body Kenyon cell responses

Our first model, “DISTINCT”, uses excitatory and inhibitory connections from the lobula orientation-sensitive type A and type B neurons originating from each quadrant of the pattern, representing the equivalent dorsal and ventral visual fields of the bees left and right eyes, respectively (see Fig 1). This allowed us to evaluate discrimination and generalisation performance of visual patterns based on these lobula neurons alone. We used 86 different types of simple excitatory and inhibitory synaptic configurations of the lobula orientation-sensitive neurons to Kenyon cells to achieve the 25°–30° orientation acuity reported for honeybees during dual trial discrimination tasks [43] (see Table 1 for Kenyon cell synapse configurations). The lobula neuron to Kenyon cell synaptic weights were fixed at +1 for the excitatory synapses, and at -1 for the EAI inhibitory synapses, such that the ± synaptic value of each Kenyon cell’s synapse would be the same as the single lobula orientation-sensitive neuron’s firing rate to which it connects (with a small amount of noise applied, see below). This model could have just as easily been configured to receive, for example, just one type B input with a synaptic weight of +3, which would have produced the exact same effect as three excitatory lobula orientation-sensitive neuron type B inputs (Fig 1). However, to reinforce the importance that there is no learning in our models, and to focus the investigation into the lobula neuronal responses, here we restrict the models to the most basic synaptic configuration, with all synaptic weights equal to ±1. The model had 30 copies of each of these Kenyon cell configuration types per quadrant, resulting in a total of 10,320 Kenyon cells, which is still a small proportion of the 340,000 Kenyon cells in the honeybee mushroom bodies [44].

**Table 1:** Lobula orientation-sensitive neuron to Kenyon cell configuration types.

001: 1A+, 1B- 002: 1A+, 2B- 003: 1A+, 3B- 004: 1A+, 5B- 005: 1A+, 7B- 006: 1A+, 11B- 007: 1A+, 13B- 008: 2A+, 1B- 009: 2A+, 3B-	010: 2A+, 5B- 011: 2A+, 7B- 012: 2A+, 11B- 013: 2A+, 13B- 014: 3A+, 1B- 015: 3A+, 2B- 016: 3A+, 5B- 017: 3A+, 7B- 018: 3A+, 11B-	019: 3A+, 13B- 020: 5A+, 1B- 021: 5A+, 2B- 022: 5A+, 3B- 023: 5A+, 7B- 024: 5A+, 11B- 025: 5A+, 13B- 026: 7A+, 1B- 027: 7A+, 2B-	028: 7A+, 3B- 029: 7A+, 5B- 030: 7A+, 11B- 031: 7A+, 13B- 032: 11A+, 1B- 033: 11A+, 2B- 034: 11A+, 3B- 035: 11A+, 5B- 036: 11A+, 7B-	037: 11A+, 13B- 038: 13A+, 1B- 039: 13A+, 2B- 040: 13A+, 3B- 041: 13A+, 5B- 042: 13A+, 7B- 043: 13A+, 11B-
044: 1A-, 1B+ 045: 1A-, 2B+ 046: 1A-, 3B+ 047: 1A-, 5B+ 048: 1A-, 7B+ 049: 1A-, 11B+ 050: 1A-, 13B+ 051: 2A-, 1B+ 052: 2A-, 3B+	053: 2A-, 5B+ 054: 2A-, 7B+ 055: 2A-, 11B+ 056: 2A-, 13B+ 057: 3A-, 1B+ 058: 3A-, 2B+ 059: 3A-, 5B+ 060: 3A-, 7B+ 061: 3A-, 11B+	062: 3A-, 13B+ 063: 5A-, 1B+ 064: 5A-, 2B+ 065: 5A-, 3B+ 066: 5A-, 7B+ 067: 5A-, 11B+ 068: 5A-, 13B+ 069: 7A-, 1B+ 070: 7A-, 2B+	071: 7A-, 3B+ 072: 7A-, 5B+ 073: 7A-, 11B+ 074: 7A-, 13B+ 075: 11A-, 1B+ 076: 11A-, 2B+ 077: 11A-, 3B+ 078: 11A-, 5B+ 079: 11A-, 7B+	080: 11A-, 13B+ 081: 13A-, 1B+ 082: 13A-, 2B+ 083: 13A-, 3B+ 083: 13A-, 5B+ 085: 13A-, 7B+ 086: 13A-, 11B+

List of all 86 lobula large-field orientation-sensitive neurons (LOSNs) to mushroom body Kenyon cell configurations. Format [configuration ID]: [number of LOSN type A synapses]A[+/- = excitatory/inhibitory synapses], [number of LOSN type B synapses]B[+/- = excitatory/inhibitory synapses]. The first 43 configurations each had one or more LOSN type A excitatory connection and one or more LOSN type B inhibitory connection. The second 43 configurations were the reciprocal of these with type A inputs being inhibitory and type B excitatory. The use of prime numbers provided a simple way to exclude duplicate responses i.e. 2A+, 5B- would generate the same Kenyon cell response as 4A, 10B-. All synaptic weights were set to 1 or -1 for the individual excitatory and inhibitory connections respectively.

The theoretical Kenyon cell connections defined above (Table 1) will each fire for a large number of perceived edge orientations and edge lengths. However, the combinatorial firing code of these 86 types allows small ranges of orientations to be uniquely identified by our models, and furthermore these edge orientations can be recognised invariant of the presented edge lengths since an almost identical combinatorial code of the fired Kenyon cells is produced if the same edge orientations are presented (see below). Adding additional lobula orientation-sensitive neuron combinations would not increase the models ability to discriminate more specific angles, as the acuity is fundamentally constrained by the particular lobula neuron response curves, which often have the same firing rate for several adjacent orientations (Fig 5). It is most likely that within the honeybee mushroom bodies a large variety of random lobula neuron to Kenyon cell synaptic connections are initially established. Equally these synapses are almost certainly plastic, adapting the synaptic strengths, and even adding and removing lobula neuron synapses, during a bee’s foraging life [45]. In this way these Kenyon cells could become highly selective and fire only for particular rewarding visual inputs. In addition, the honeybee brain may be capable of adjusting the Kenyon cell synapse strengths to better account for noise in the lobula orientation-sensitive neuron responses and produce more effective combinatorial codes for identifying particular orientations than our models (see Discussion). However, since this study is primarily concerned with the lobula orientation-sensitive neurons effectiveness as feature detectors and their affect on the honeybees’ ability to discriminate and generalize achromatic patterns, and not on learning or other ‘fine-tuning’ neuronal mechanisms, this additional model complexity of random connectivity and weight adaption was omitted.

Each models’ Kenyon cell response, to a given pattern, was calculated by first summating the value of all its synapses (number and type of synapses dependent on that Kenyon cells particular configuration type (Table 1)). If this total summated synaptic input was greater than zero the output of the Kenyon cell was set to 1 (fired). Otherwise the response was set to 0 (completely inhibited). The individual Kenyon cell synaptic values were calculated by taking the firing rate of the connected lobula orientation-sensitive neuron, plus a small synaptic signal to noise distortion, and multiplying this by +1 for excitatory synapses and -1 for inhibitory ones. The noise was added to account for natural variation in both the lobula orientation-sensitive neurons’ responses when presented with the same pattern, and in pre- and post- synaptic neurotransmitter signals. Matlab’s (Matworks) AWGN (add white Gaussian noise to signal) function was used with a signal to noise ratio value of 30. This setting produced approximately 2–5Hz variations on the 36Hz response of the type A lobula orientation-sensitive neuron at its maximal sensitivity and an edge length of 280 pixels. This would be similar to the response variation reported in the honeybee lobula neurons after the deduction of the neuronal background firing rates [13]. In this way the binary values of all 10,320 Kenyon cell responses were calculated; these values were stored in an array and saved cross-referenced to the pattern’s UID.

Given the apparent non-retinotopic distribution of visual inputs from the corresponding left and right eye regions in the bee mushroom bodies [16] the second model “MERGED” was created to explore the effect of merging lobula orientation-sensitive neuron synaptic connections from both eyes onto the Kenyon cells. To keep our theoretical model simple and comparable to the DISTINCT model, we again relied on the 86 lobula neuron to Kenyon cell configuration types (Table 1). However, in this model, rather than the previous model’s segregation of Kenyon cells into different groups per quadrant, here just two distinct groups of Kenyon cells were formed; one group of Kenyon cells all received lobula orientation-sensitive neuron type A and type B inputs from the dorsal regions of both left and right eyes, and a similar group of Kenyon cells receiving the four lobula inputs from the ventral regions of the eyes (see Fig 1). This MERGED model again had 30 copies of each configuration type, which created in total 5,160 Kenyon cells.

### Pre-processing of patterns

Each achromatic pattern used in this study was taken from the pdf document of the published behavioural papers. These images were scaled and centred to fit within a 150 x 150 pixel PNG image. Where pattern image resolution was insufficient, we recreated the patterns in Microsoft PowerPoint using the stimuli instructions provided in the papers’ method sections. For the offset discrimination experiments, the 150 x 150 pixel patterns were placed centrally within a larger white 300 x 150 pixel image and horizontally offset left and right between 0 and 200 pixels in 25 pixel increments to create a set of 17 test images per original pattern. For offsets greater than 75 pixels the original images were cropped accordingly (see Fig 2).

All images were processed in Matlab (Mathworks) in the following way:

- Removal of excess pixel noise in the image
- Conversion to a binary black and white image using only the green channel
- Calculation of the orientation and gradient magnitude of each edge in the image using Canny edge detection and Sobel gradient analysis
- Removal of short edges with a gradient magnitude ≤ 1.7. Equivalent to those edges subtending less than 3° on a honeybee eye, which have been shown to be undistinguishable by bees [46, 47]
- Division of the image into four equal quadrants; for each quadrant we generated a histogram analysis of all oriented edge lengths in 1° increments (0°-180°)
- Saving the histogram dataset for each quadrant into a unique file per image

### Calculating Kenyon cell similarity ratios and experimental performances

**Fig 7:**
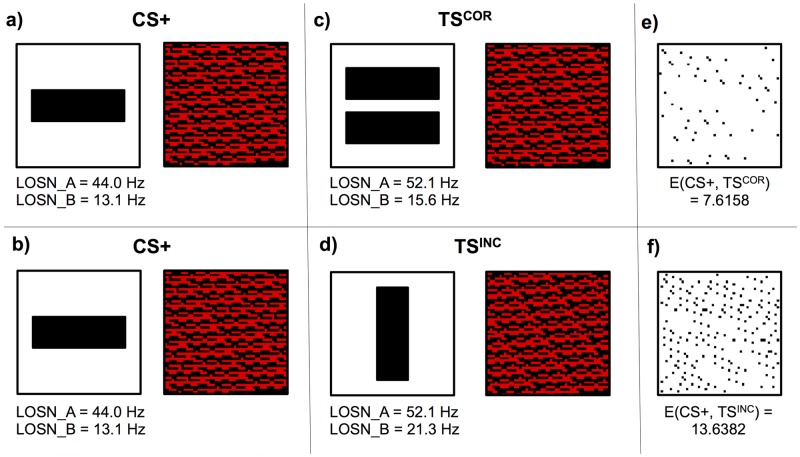
Worked example of LOSN firing rate response calculations. Simplified example of the lobula orientation-sensitive neuron (LOSN) type A and type B firing rate response calculations. **(a, b)** Left: rewarding pattern (CS+) (single horizontal bar). Here we used the DISTINCT model to calculate the Kenyon cell activations to this pattern. Right: graphical representation of all Kenyon cell activations (red: fired, black: inhibited). In these example we again only processed the top-left quadrant of the visual field (see Fig 6). **(c)** The correct test stimulus (TS^COR^) and the resultant Kenyon cell activations. **(d)** Incorrect test stimulus (TS^INC^) and its Kenyon cell activation pattern. **(e)** Black dots show if differences occur between the activation of respective Kenyon cells when presented with rewarding pattern and correct test patterns. **(f)** Differences between the rewarding and incorrect stimuli Kenyon cell activations. The rewarding (CS+) and correct test stimuli (TS^COR^) both present mostly horizontal edges; however due to the difference in edge lengths, the lobula orientation-sensitive neuron firing rates are markedly different. Nonetheless, the combination of excitatory and inhibitory synaptic connections from these lobula neurons to the Kenyon cells (see Table 1) produces very similar Kenyon cell activations. Using the Euclidian distances between the Kenyon cell activations of the CS+ and TS^COR^, and CS+ and TS^INC^ responses this simulation produced a Kenyon cell similarity ratio of (1 − (7.6158 / (7.6158 + 13.6382))) = 0.64 (see Eq 2); indicating that for this simulation our DISTINCT model would generalize from the single horizontal bar pattern (CS+) to the two horizontal bars pattern (TS^COR^), in preference to the single vertical bar stimulus (TS^INC^).

Each experiment simulated in this study was composed of three patterns, the rewarding pattern (CS+) used during the honeybee training and two novel test patterns used in the experimental evaluation trial. The test stimuli patterns that honeybees preferred during their trials were designated as correct test stimuli (TS^COR^) and the incorrect test stimuli (TS^INC^) were accordingly the patterns the bees least preferred. To simulate the experiments from published behavioural work, we first pre-processed the lobula orientation-sensitive neuron responses for all the used patterns and compiled them in an experiment-specific unique Matlab (Mathworks) file (hereafter referred to as “study file”). For each individual experiment within a study file, we defined the CS+, TS^COR^ and TS^INC^ pattern unique identifiers (UIDs) as well as recoding the behavioural results of the honeybees. For each model we loaded the study file, extracted the unique pattern image IDs for each experiment and the corresponding eight lobula neuron firing rate values and from these calculated the model’s Kenyon cell responses to all three patterns. This provided separate arrays of binary Kenyon cell responses for the three patterns (rewarding stimulus, correct test stimulus and incorrect test stimulus), which we used to calculate the Euclidian distance from the rewarding stimulus array to the correct stimulus array, and rewarding stimulus array to the incorrect stimulus array (see Fig 7). The ratio of these two Euclidian distances produced a Kenyon cell similarity ratio for that experiment for a single simulation trial (Eq 2). Each experimental simulation was repeated one thousand times and the average, standard deviation, minimum and maximum Kenyon cell similarity ratio results of each experiment were recorded.

$$KCSR=\;1-\;\frac{\text{E}\left(\text{CS}+,\text{TSCOR}\right)}{\text{E}\left(\text{CS}+,\text{TSCOR}\right)+\text{E}\left(\text{CS}+,\text{TSINC}\right)}$$ (2)

Where KCSR: Kenyon cell similarity ratio; E(x, y): Euclidian distance between x and y; CS+, TSCOR, TSINC: array of Kenyon cell response values for the respective patterns.

For the generalisation experiments, honeybees had been trained on multiple rewarding and unrewarding pattern pairs selected from relevant pools (Fig 3) [19, 20]. We followed the same procedure as above but created individual simulations for each possible pattern triplet combination. As the behavioural results also included the choice selections of different groups of bees trained on the reciprocal of the learned association (i.e. rewarding patterns became unrewarding patterns, and vice versa), we used the published unrewarding training patterns as a new set of rewarding (CS+) simulation patterns and paired them with the according correct and incorrect test patterns. Simulations were again performed one thousand times for all pattern triplet combinations. The Kenyon cell similarity ratio results for all combinations were then averaged to create an overall Kenyon cell similarity ratio value for that particular pattern test.

Due to the difficulties attaining electrophysiological recordings from honeybees during visual learning tasks [41, 48–50] we know almost nothing about how a bee’s final behavioural decision is underpinned by neuronal firing patterns in the visual system or mushroom bodies. However, we can assume that if the Kenyon cell responses to a presented test stimulus are very similar to those generated by a previously learnt rewarding training stimulus (i.e. the same pattern is presented) and the distractor pattern is very different to the learnt rewarding pattern, then the honeybee Kenyon cell similarity ratio would be almost 1.0, and we would expect the bee to almost always visit the correct test pattern, with an experimental correct choice performance close to 100%. Similarly, if the correct and incorrect test patterns are different from each other and also different to the learnt rewarding pattern, but both produced Kenyon cell responses equally similar/dissimilar to that of the rewarding pattern (i.e. Kenyon cell similarity ratio = 0.5) then we would expect the honeybee to visit each pattern equally likely, and therefore over multiple trials (and multiple bees) have an experimental ‘correct’ choice performance of approximately 50%. Furthermore, if the honeybees were trained on a particular rewarding pattern and then tested with a correct test pattern similar to this learnt stimulus and a very different incorrect test pattern, and then a second test conducted with the same correct pattern and a very similar incorrect pattern, we would again assume the honeybees correct choice accuracy for the first test would be far higher than the second test. Similarly, the Kenyon cell similarity ratio of the first experiment would undoubtedly be much higher than that of the Kenyon cell similarity ratio of the second experiment.

Consequently, to allow us to compare our model simulation results directly against the empirical honeybee experimental results we make the following assertion: our models’ simulated bee performances for any given experiment are directly correlated to the average Kenyon cell similarity ratio of all simulation trials for that experiment. In this way if a model’s average Kenyon cell similarity ratio for a given experiment were 0.64 then its simulated bee’s overall experimental performance for selecting the correct test pattern would be 64%. It would have been possible to implement a probabilistic ‘Monte Carlo’ style binary response for the simulated bees to choose either the corrector incorrect test pattern per trial (based on that simulation trial’s Kenyon cell similarity ratio result) and subsequently calculate the proportion of correct choices (as with honeybees). However, this would have added probabilistic variability, whereas the Kenyon cell similarity ratio values are variant on just the small amount of synaptic noise applied to the lobula orientation-sensitive neuron to Kenyon cell connections (which is biologically relevant), therefore this additional probabilistic step was judged an unnecessary and potentially detrimental complication. The above assertion does have some limitations when assuming a direct comparable mechanism within the honeybee brain (see Discussion), but nonetheless this provides an effective method for assessing how the lobula orientation-sensitive neuron responses, as well as their Kenyon cell connection configurations, affect the models’ performances over a wide range of pattern experiments. This mechanism also benefits from not needing to train and test an artificial neural network on each pattern experiment, and the inherent parameter tuning and subsequent performance evaluations that this approach would require.

It would have been desirable to assess how our models correlated with the honeybees’ relative performances over all of the tested experiments. Each set of the original honeybee generalisation experiments [19, 20] only provided a number of mean data points for comparison. In each study, the bees were tested on patterns that typically varied in one particular aspect (e.g. number and orientation of bars in each pattern quadrant), but were similar otherwise. Moreover, the used publications addressed similar issues and used similar patterns. While this is a good approach when probing the limits of the learning abilities of bees, it also means that the data points are not independent due to pseudo-replication. A correlation coefficient involving data from multiple different experiments would, therefore, be misleading. Instead, we displayed our simulated bee experimental performance results (equivalent to the Kenyon cell similarity ratio averages over all of that experiment’s simulations) side-by-side with the empirical data. These were grouped into five batches of related generalization tasks, similar to that done in the original studies, so that the relative performance of the different simulated experiments could be assessed, and compared to that of the real honeybees’ relative performances on the same sets of pattern pairs.
